# Residual Stress Measurement Using EMAT for X80 Pipeline Steel: Effects of Coating Thickness and Surface Roughness Under Low Surface Preparation Requirements

**DOI:** 10.3390/ma17235799

**Published:** 2024-11-26

**Authors:** Chunlang Luo, Bing Chen, Li Xia, Lintao Xu, Xuan Liu, Sunmin Zou, Dongchuan Peng, Guoqing Gou

**Affiliations:** 1 Key Laboratory of Advanced Technologies of Materials (Ministry of Education), School of Materials Science & Engineering, Southwest Jiaotong University, Chengdu 610031, China; 2 Zhejiang Academy of Special Equipment Science, Hangzhou 310009, China

**Keywords:** EMAT, residual stress, X80 pipeline steel, coating thickness, surface roughness

## Abstract

The residual stress significantly affects the operational safety of oil and gas pipelines. Traditional ultrasonic stress measurement methods require pipeline surface pretreatment, which reduces detection efficiency. EMAT, as a non-contact measurement method, shows promising applications for residual stress detection in oil and gas pipelines. Therefore, based on field conditions for residual stress detection in oil and gas pipelines, this study prepared X80 pipeline steel specimens with epoxy resin coatings of 0.58 mm, 1 mm, 1.58 mm, and 1.9 mm thickness to verify the influence of coating thickness on the stress measurement accuracy of EMAT. Additionally, X80 pipeline steel specimens with varying surface roughness were prepared to study the impact of surface roughness on the residual stress measurement. The results indicate that within the range of coating thickness variations, the residual stress measurement error falls in the range of 50 MPa, while the change of residual stress caused by surface roughness is less than 30 MPa. This validates the feasibility and accuracy of the EMAT method for residual stress measurement in in-service pipelines without the need for surface treatment.

## 1. Introduction

Oil and gas pipelines, due to their complex structure, diverse welding methods, and harsh service environments, are susceptible to substantial local residual stresses during welding, transportation, and service [1,2,3]. Excessive residual stress weakens pipeline structures, potentially leading to pipeline failure and safety accidents in severe cases [4]. Consequently, detecting residual stress in pipelines is crucial both after welding and during service. Residual stress detection methods are broadly categorized into destructive and non-destructive testing (NDT) methods [5]. Destructive testing damages the structure of components, rendering it unsuitable for in-service applications, whereas NDT effectively measures residual stress in components without interruption of service. An NDT method, ultrasonic testing, based on the acoustoelastic effect is widely employed for residual stress detection in welded structures due to its non-destructive, efficient, and high-precision characteristics [6,7]. The longitudinal critically refracted (LCR) wave technique is widely utilized in pipeline stress detection. However, as a contact measurement, its accuracy is highly sensitive to coupling conditions and surface roughness [8,9]. Welded pipeline surfaces are typically rough with oxide layers, and in-service pipelines are often coated to a certain thickness. The LCR wave technique generally requires surface grinding to accurately measure pipeline residual stress, significantly reducing inspection efficiency. In contrast, an electromagnetic acoustic transducer (EMAT), operating based on Lorentz force or magnetostrictive mechanisms, offers a non-contact measurement method. EMAT employs electromagnetic coupling to excite and receive ultrasonic waves, requiring minimal surface preparation, and no coupling agent, and can operate in high-temperature environments [10]. These features make EMAT particularly suitable for on-site residual stress detection in pipelines. Thus, EMAT demonstrates a strong potential for residual stress detection in oil and gas pipelines.

Currently, the EMAT is applied in areas such as wall thickness measurement, damage assessment, and corrosion detection in oil and gas pipelines [11,12,13]. Thon et al. [14] used a linear array EMAT combined with phase velocity excitation technology to inspect a steel pipe with a diameter of 323.8 mm and a wall thickness of 10.2 mm. The results showed that this method could locate defects within a propagation distance of 600 mm, with an error under 10 mm, and a minimum thickness measurement error of less than 0.4 mm. Song et al. [15] proposed a novel method that combined EMAT and eddy current techniques for detecting internal and external corrosion defects in pipelines. This method utilizes time-division multiplexing to separate mixed signals and extract independent features for diagnosing potential pipeline defects, offering significant advantages in detectability and efficiency over traditional methods. However, there is relatively little research on using EMAT for residual stress detection, particularly in the stress detection of oil and gas pipelines. Murav’ev et al. [16] used EMAT based on the acoustoelastic effect to measure stress in R65 rail steel. The experimental results were highly consistent with theoretical values, showing that an increase in rail temperature decreases tensile stress, while a temperature decrease has the opposite effect. Wang et al. [17] applied EMAT to measure residual stress in Q345 steel plates and compared the results with numerical simulations. The findings showed that EMAT measurements were consistent with simulations along the weld direction, but significant errors appeared perpendicular in the perpendicular direction and next to the fusion line. Dang et al. [18] proposed a new non-contact method based on a dual-mode EMAT for residual strain detection in metal structures, which does not require prior knowledge of thickness. Their EMAT system enables a quantitative, non-destructive measurement of longitudinal and shear-wave velocities in uniaxially stretched aluminum plates under varying residual strains. All in all, EMAT demonstrates considerable potential in residual stress detection for oil and gas pipelines. However, further research is still needed to improve its detection accuracy and stability under complex operating conditions.

This study first derives a theoretical formula for residual stress measurement using the EMAT shear-wave birefringence method based on acoustoelastic theory. Subsequently, the self-developed EMAT equipment is used to measure the residual stress in X80 pipeline steel specimens with varying coating thicknesses to investigate the effect of coating thickness on measurement accuracy. In addition, an error analysis of residual stress measurements is conducted on X80 pipeline steel with varying surface roughness to assess the impact of surface roughness on stress measurement. The research findings provide a reference for the practical application of EMAT in pipeline residual stress measurements, including in scenarios such as elbows, bends, valves, and other components.

## 2. Stress Detection Theory of EMAT

**Figure 1:**
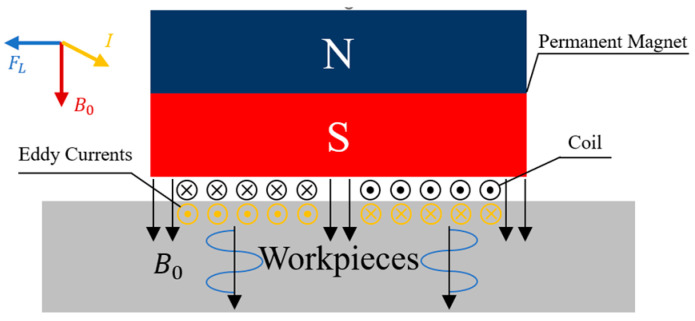
Schematic diagram of EMAT.

The operating principle of EMAT is based on electromagnetic induction. When a high-frequency coil is placed on the surface of a workpiece and energized with high-frequency current, eddy currents are induced within the skin depth region of the workpiece surface. These eddy currents interact with the external static magnetic field, experiencing Lorentz forces. Under alternating stress, the metal medium generates stress waves within the ultrasonic frequency range. Conversely, due to the reversibility of this effect, when ultrasonic wave echoes from within the workpiece reach the surface, the particles of the near-surface of the workpiece are vibrated. In this state, the high-frequency coil generates a voltage under the magnetic field, producing an electrical signal that can be received [19,20]. The schematic diagram is shown in Figure 1. The generation and propagation of EMAT occurs entirely within the workpiece, making it a non-contact measurement method capable of detecting stress at a certain lift-off distance from the transducer to the workpiece. This technique eliminates the need for traditional couplings, making it suitable for stress detection in metallic materials under complex conditions.

Assuming the sample is isotropic and under plane stress conditions, $\sigma_{11}=0$, $\sigma_{22}\neq 0$, and $\sigma_{33}\neq 0$, the shear waves propagate along the thickness direction of the sample. The relationship between the shear-wave velocities $v_{12}$ and $v_{13}$ in the two perpendicular directions of the plane and the stresses $\sigma_{22}$ and $\sigma_{33}$ is expressed by the following formula [21]:

(1) $$\rho_{0}v_{12}^{2}=\mu +\frac{\sigma_{22}}{3K_{0}}(\lambda +2\mu +m+\frac{\lambda n}{4\mu })+\frac{\sigma_{33}}{3K_{0}}(m-2\lambda -\frac{\lambda +\mu }{2\mu }n)$$

(2) $$\rho_{0}v_{13}^{2}=\mu +\frac{\sigma_{33}}{3K_{0}}(\lambda +2\mu +m+\frac{\lambda n}{4\mu })+\frac{\sigma_{22}}{3K_{0}}(m-2\lambda -\frac{\lambda +\mu }{2\mu }n)$$

In the equation, $\rho_{0}$ represents the material density under zero-stress conditions, $v_{12}$ and $v_{13}$ are the ultrasonic wave velocities propagating along coordinate axes 2 and 3, respectively, λ and μ are the second-order elastic Lame constants, and m and n are the third-order elastic Murnaghan constants.

From the Equations (1) and (2), the ultrasonic shear-wave velocity $v_{0}$ under zero stress conditions is given by the following equation:

(3) $$v_{12}\approx v_{13}\approx v_{0}=\sqrt{\frac{\mu }{\rho_{0}}}$$

The procedure for substituting Equation (3) into Equations (1) and (2) is as follows:

(4) $$\frac{v_{12}-v_{0}}{v_{0}}=\frac{\sigma_{22}}{3K_{0}\cdot 2\mu }C_{1}+\frac{\sigma_{33}}{3K_{0}\cdot 2\mu }C_{2}$$

(5) $$\frac{v_{13}-v_{0}}{v_{0}}=\frac{\sigma_{33}}{3K_{0}\cdot 2\mu }C_{1}+\frac{\sigma_{22}}{3K_{0}\cdot 2\mu }C_{2}$$

In the equation, $C_{1}=\frac{\lambda +2\mu +m+\frac{\lambda n}{4\mu }}{3K_{0}\cdot 2\mu }$, $C_{2}=\frac{m-2\lambda -\frac{\lambda +\mu }{2\mu }n}{3K_{0}\cdot 2\mu }$.

By adding and subtracting Equations (4) and (5), an equation is obtained that describes the relationship between the speed of sound and stress in an isotropic material under a plane stress state:

(6) $$\frac{v_{12}-v_{13}}{v_{0}}=A(\sigma_{22}-\sigma_{33})$$

(7) $$\frac{v_{12}+v_{13}-2v_{0}}{v_{0}}=B(\sigma_{22}+\sigma_{33})$$

In the equation, $A=C_{1}-C_{2}$, $B=C_{1}+C_{2}$.

The material structure varies in different directions, leading to unequal transverse wave velocities in all directions. Therefore, it is necessary to modify Equations (4) and (5), and the corrected relationship between the speed of sound and stress in the anisotropic material is as follows:

(8) $$\frac{v_{12}-v_{13}}{v_{0}}=\alpha +A\sigma_{22}+B\sigma_{33}$$

(9) $$\frac{v_{12}+v_{13}-2v_{0}}{2v_{0}}=C\sigma_{22}+D\sigma_{33}$$

The tensile machine accuracy verification test involves unidirectional stress, where parameters $\sigma_{11}=0$, $\sigma_{22}\neq 0$, and $\sigma_{33}=0$, reduce Equations (8) and (9) to the following:

(10) $$\sigma_{22}=K\frac{v_{12}-v_{13}}{(v_{12}^{0}+v_{13}^{0})/2}+\alpha$$

The transverse echo propagation time can be obtained from the test, and, using Equation (10), an accurate measurement of the sample thickness is required. Therefore, Equation (10) is transformed to yield Equation (11):

(11) $$\sigma_{22}=K\frac{t_{12}-t_{13}}{\left(t_{12}^{0}+t_{13}^{0}\right)/2}+\alpha$$

where K is the stress coefficient, $\frac{t_{12}-t_{13}}{\left(t_{12}^{0}+t_{13}^{0}\right)/2}$ is the anisotropy coefficient, $t_{12}^{0}$ is the shear-wave propagation time parallel to the stress loading direction, and $t_{13}^{0}$ is the shear-wave propagation time perpendicular to the stress-loading direction.

## 3. Experimental Contents

### 3.1. Experimental Equipment

**Figure 2:**
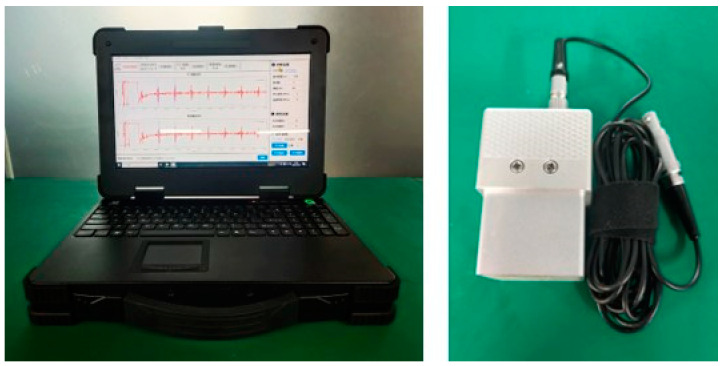
Residual stress measurement equipment and transducer of EMAT.

The experiment deployed a portable EMAT device independently developed by Southwest Jiaotong University (Chengdu, China). This device can generate excitation pulse signals of up to 1500 Vpp, with a peak power of 15 kW. The width and number of ultrasonic excitation pulses are adjustable, and the gain range of the echo signal is from 20 dB to 100 dB. The EMAT transducer has a center frequency of 3.8 MHz. The specific appearance of the equipment is shown in Figure 2.

### 3.2. Calibration Process

The EMAT-based stress measurement is based on the theory of acoustic elasticity, which states that the propagation speed of ultrasonic waves in a given medium is related to the internal stress state of the material. The calibration process establishes a relationship model between stress and sound velocity by measuring the ultrasonic wave speed under known stress conditions.

**Figure 3:**
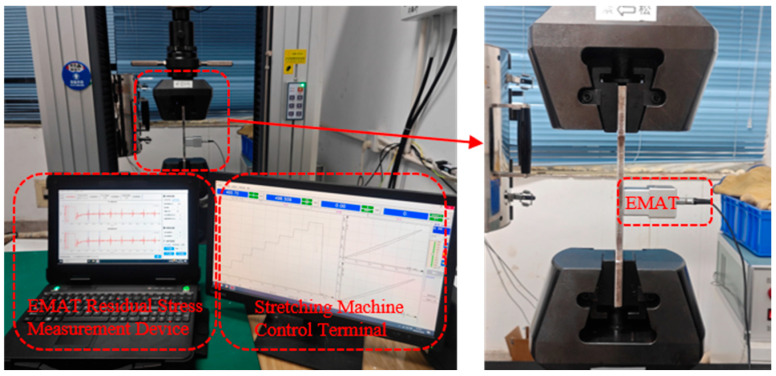
Stress-acoustic anisotropy factor calibration process.

The calibration procedure was as follows: (1) Install the X80 pipeline steel calibration specimen on the tensile testing machine. (2) Position the electromagnetic ultrasonic transducer at the center of the calibration specimen. (3) Use the tensile testing machine to apply incremental loads to the calibration specimen, ranging from 0 MPa to 400 MPa in 50 MPa increments. At each 50 MPa increment, measure the echo signal using the EMAT, calculate the parallel and perpendicular polarization times, and repeat the tensile test three times. (4) At each step, calculate the acoustic anisotropy factor using the parallel and perpendicular polarization times. (5) Use linear curve fitting to obtain the specific expressions for the parameters K and α in the equation. The tensile calibration process is shown in Figure 3.

### 3.3. Coating Specimen Preparation

As a non-contact detection method, EMAT relies on electromagnetic induction between the high-frequency coils in the transducer and the near surface of the workpiece to propagate the ultrasonic excitation and echo signals. The closer the transducer is to the workpiece surface, the stronger the ultrasonic excitation and echo signals become [22,23]. However, the presence of the coating increases the distance between the transducer and the workpiece surface, weakening the echo signal strength. Additionally, the coating may increase the attenuation of the ultrasonic waves during transmission, further reducing the sensitivity of residual stress measurements. The effect of the proximity of the transducer to the tested material on the strength of the ultrasonic echo signals can be described by the following formula:

(12) $$S(g)=S_{0}e^{\left(\frac{-2\pi g}{D}\right)}$$

where S represents the function of signal intensity varying with the lift-off distance; $S_{0}$ denotes the signal intensity when the contact gap is zero between the transducer and the workpiece; g stands for the distance between the coil and the surface of the workpiece; and D indicates the distance between the coils within the transducer.

**Figure 4:**
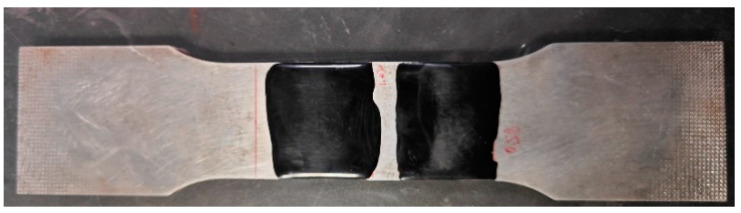
Coating samples.

To verify the influence of the coating thickness on the accuracy of stress measurements using EMAT, this study applied epoxy resin of varying thicknesses (0.58 mm, 1 mm, 1.58 mm, and 1.9 mm) to X80 pipeline steel tensile samples, reflecting the surface conditions of X80 pipeline steel in service. The details of the coated samples are shown in Figure 4. A tensile testing machine was used to apply stress to the samples with different coating thicknesses, starting from 0 MPa and gradually increasing by 50 MPa increments until reaching 400 MPa. Stress measurements were taken at the same location on the coated samples under different loads to obtain the stress values and corresponding waveforms of the echo signal using EMAT.

### 3.4. Roughness Specimen Preparation

Currently, the primary method for stress detection in pipelines is the (LCR) wave technique. When measuring residual stress using the LCR method, a coupling agent must be applied between the ultrasonic transducer and the specimen surface to ensure proper coupling conditions, which are essential for both transmitting and receiving LCR waves [9]. The LCR wave propagates along the near surface of the sample, where surface roughness not only impacts the coupling condition but also interferes with the propagation of the LCR wave, thus directly affecting the accuracy of stress measurements.

Surface roughness also affects the accuracy of wall thickness measurements using EMAT. As the surface roughness increases, the average Lorentz force density decreases, reducing the excitation energy of EMAT [24]. Additionally, a rough surface generates more interfering sound waves, which significantly attenuate the signal-to-noise ratio (SNR), thereby impacting measurement accuracy [25]. Therefore, this study systematically investigates the effect of surface roughness on residual stress measurements using both LCR and EMAT methods by preparing specimens with varying levels of surface roughness.

**Figure 5:**
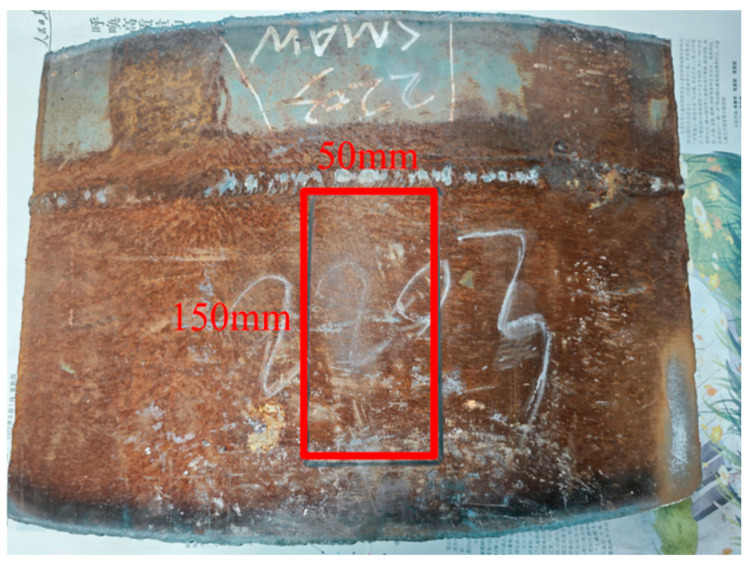
X80 pipeline steel specimen on post-service.

The annular butt weld sample was cut from X80 pipeline steel after service (Figure 5). The red-marked area of the sample (150 mm × 50 mm) was polished at a low speed using 600-grit sandpaper. After every 40 polishing cycles, residual stress measurements were conducted using EMAT and LCR at nine locations: 30 mm, 45 mm, 60 mm, 75 mm, 90 mm, 105 mm, and 120 mm from the weld to study the effect of polishing frequency on residual stress measurement.

**Figure 6:**
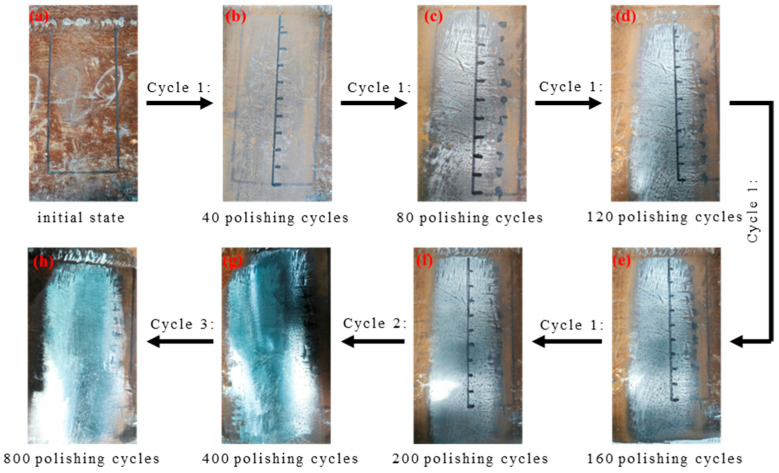
X80 pipeline steel roughness specimen. (**a**–**h**) represent the surface conditions of roughness samples that are unpolished and polished 40 times, 80 times, 120 times, 160 times, 200 times, 400 times, and 800 times, respectively.

Figure 6 illustrates the polishing process of X80 pipeline steel, where Cycle 1, Cycle 2, and Cycle 3 correspond to 40, 200, and 400 polishing cycles, respectively. Throughout the sample preparation, seven polishing steps were performed to produce eight distinct surface states: unpolished, 40-cycle polished, 80-cycle polished, 120-cycle polished, 160-cycle polished, 200-cycle polished, 400-cycle polished, and 800-cycle polished, which are represented in Figure 6a–h.

Figure 6a shows the initial unpolished state of the sample, where the pipeline steel surface is covered with substantial rust, pits, and uneven protrusions. After 80 polishing cycles (Figure 6c), the rust on the surface was removed, but the pits and unevenness caused by long-term corrosion and external impact remained difficult to eliminate entirely. With 160 polishing cycles (Figure 6e), the surface became smoother and displayed slight reflectivity. After 200 polishing cycles (Figure 6f), the surface exhibited a metallic luster, revealing the original substrate, although minor pits and fine scratches still had the potential to affect measurement accuracy. At 400 polishing cycles (Figure 6g), the larger pits were mostly removed, leaving only small residual imperfections. Finally, after 800 polishing cycles (Figure 6h), the sample surface became completely smooth and free of any remaining pits, achieving the desired surface condition.

Table 1 presents the surface roughness results measured at different surface states using the contact roughness tester. As the number of polishing cycles increased, the surface roughness progressively decreased from 180.3 μm in the unpolished state to 18.2 μm after 800 polishing cycles.

## 4. Results and Discussion

### 4.1. Calibration Results

**Figure 7:**
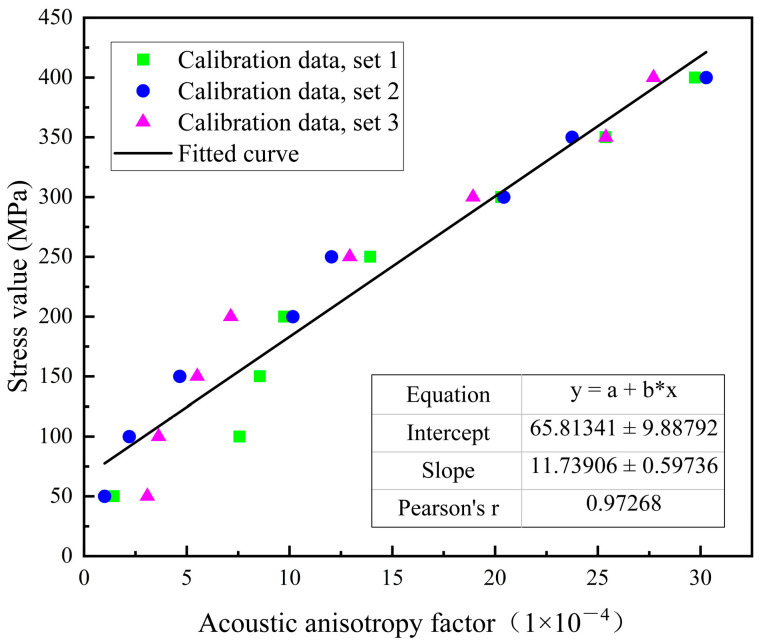
Stress-acoustic anisotropy factor calibration curve.

The calibration results of the stress-acoustic anisotropy factor were linearly fitted to establish a relationship between stress and the acoustic anisotropy factor. Figure 7 shows the linear relationship after fitting. As shown in the figure, stress and the acoustic anisotropy factor exhibit good linear correlation, indicating that the variation of the acoustic anisotropy factor follows a linear model within this stress range. Based on the fitting results, the equation describing the relationship between stress and the acoustic anisotropy factor is as follows:

(13) σ=11.7x+65.8

where σ represents the stress value measured by EMAT, and x is the acoustic anisotropy factor.

The root mean square error (RMSE), correlation coefficient (R^2^), and uncertainties of the parameters in the linear fitting model (slope and intercept) are 26.6 MPa, 0.973, 0.59, and 9.88, respectively. These results indicate that the calibration equation effectively explains the relationship between stress and signal variation under the current experimental conditions, demonstrating excellent applicability and reliability.

### 4.2. Influence of Coating Thickness

**Figure 8:**
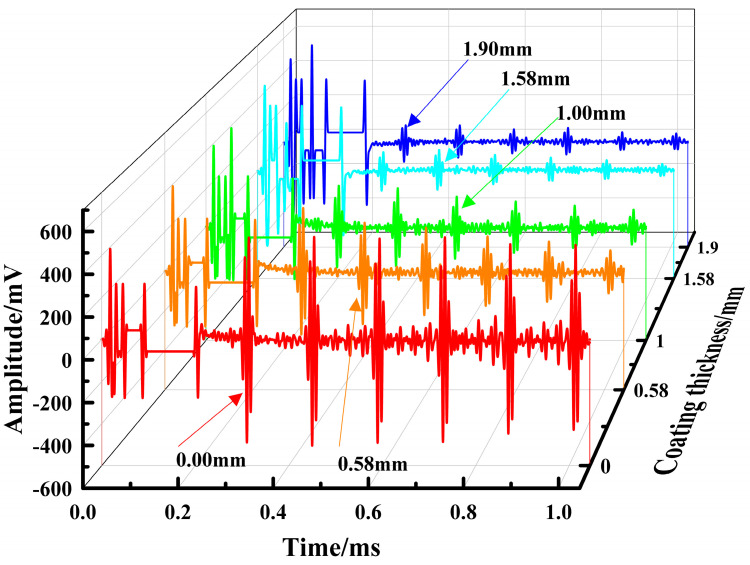
Echo signal at different coating thicknesses.

To investigate the effect of coating thickness on the accuracy of stress measurements with EMAT, stress measurements were taken on samples with different coating thicknesses and compared with the applied load. Table 2 presents the amplitudes of the first echo signal for different coating thicknesses, and Figure 8 displays the corresponding EMAT echo waveforms. To ensure testing consistency, the stress measurement gain for samples with different coating thicknesses was set to 35 dB. As the coating thickness increases, the amplitude of the echo signal gradually decreases. This result agrees with the findings of Xiang et al. [26] and Huang et al. [27] regarding the effect of EMAT surface wave lift-off distance on echo signals: as the lift-off distance between the transducer and workpiece surface increases, the echo signal amplitude exhibits an exponential attenuation, which is consistent with the theoretical relationship described in Equation (12).

**Figure 9:**
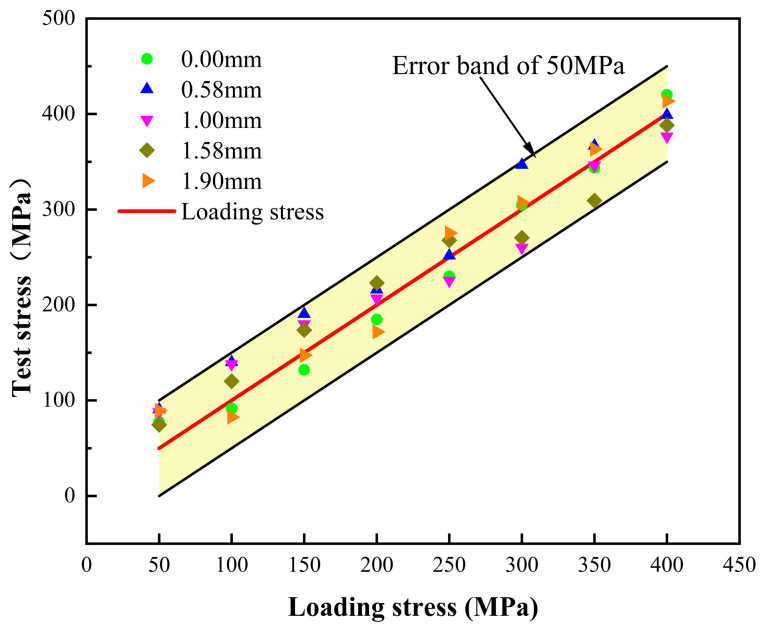
Stress accuracy verification of different coating thicknesses.

Table 3 and Figure 9 show the stress measurement values of EMAT under different coating thicknesses. The measured stress values fall within a 50 MPa error range under various coating conditions, indicating that even with an increased lift-off distance, the measurement error remains controllable within 10% of the yield strength (555 MPa) of X80 pipeline steel. This result demonstrates that EMAT can reliably measure stress under pipeline coating conditions.

The presence of the coating and its increased thickness cause the lift-off distance between the EMAT transducer and the workpiece to increase, thus reducing the amplitude of the echo signal. However, since EMAT waves primarily generate near the specimen surface and propagate along the thickness direction within the sample, they are less affected by the coating. Provided that the SNR of the echo signal is ensured, the decrease in echo amplitude caused by changes in lift-off distance has a relatively small effect on stress measurement accuracy.

### 4.3. Influence of Surface Roughness

**Figure 10:**
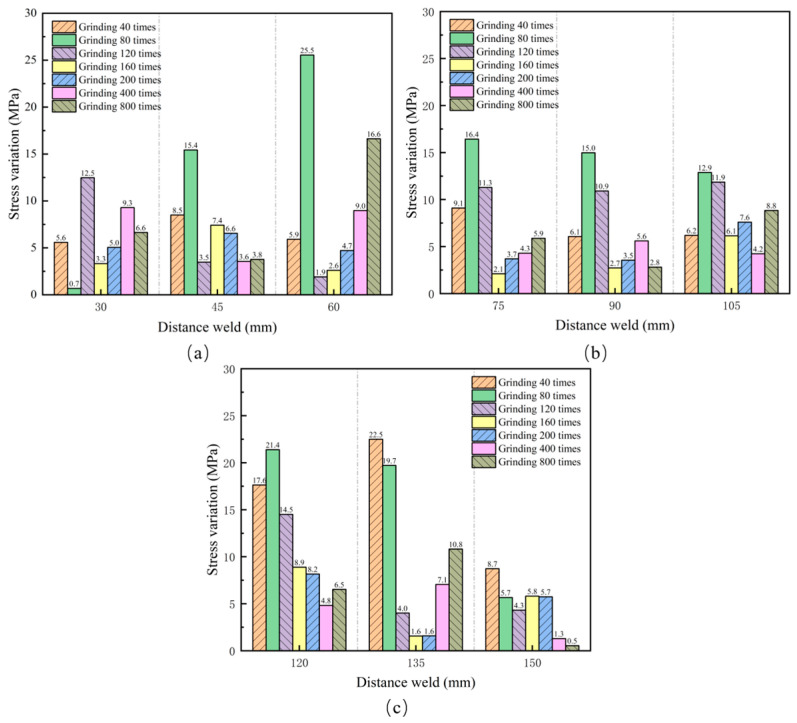
Stress variation under different polishing conditions. (**a**) Stress changes at 30, 45, and 60 mm from the weld; (**b**) stress changes at distances of 75, 90, and 105 mm from the weld; (**c**) stress changes at 120, 135, and 150 mm from the weld.

To investigate the effect of surface roughness on the accuracy of stress measurements with EMAT, residual stress measurements were taken on unpolished and polished X80 pipeline steel samples using EMAT equipment. Figure 10 shows the absolute difference between the residual stress values measured at each point after polishing and those measured in the unpolished state. The results indicate that the maximum change occurred at the position 60 mm from the weld after 80 polishing cycles, with a value of 25.5 MPa, while the minimum change occurred at a location 150 mm from the weld after 800 polishing cycles, with a value of 0.5 MPa. Overall, the residual stress variations measured by EMAT were all less than 30 MPa, indicating that the residual stress values before and after polishing showed minimal changes.

**Figure 11:**
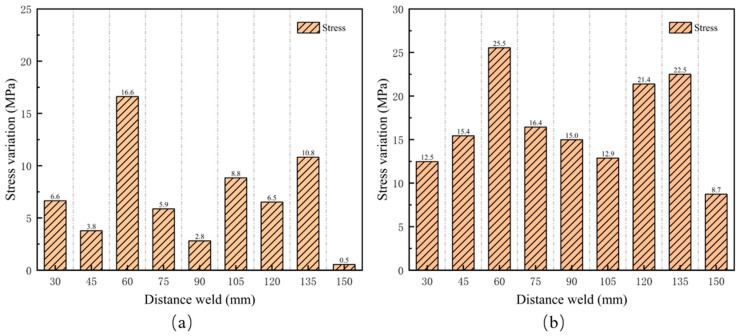
Residual stress changes before and after grinding. (**a**) Changes in residual stress after unpolished and polished 800 times; (**b**) maximum change of stress before and after grinding.

Figure 11a shows the residual stress variations measured by EMAT before and after 800 polishing cycles. The maximum change was 16.6 MPa at 60 mm from the weld, while the minimum variation was 0.547 MPa at 150 mm from the weld. In the tests, the stress value at 150 mm from the weld in the unpolished state served as the reference. After 800 polishing cycles, the variation in the test values at this position was only 0.5 MPa, indicating that the stress measurement remained largely unchanged after sufficient polishing.

Figure 11b shows the maximum variation in residual stress measured by EMAT during different polishing cycles. The maximum stress variation measured was 25.5 MPa, with an average of 16.7 MPa, and the stress variation was less than 5% of the yield strength (555 MPa) of X80 pipeline steel. Therefore, it can be concluded that polishing has a minimal effect on the accuracy of residual stress measurements using EMAT.

**Figure 12:**
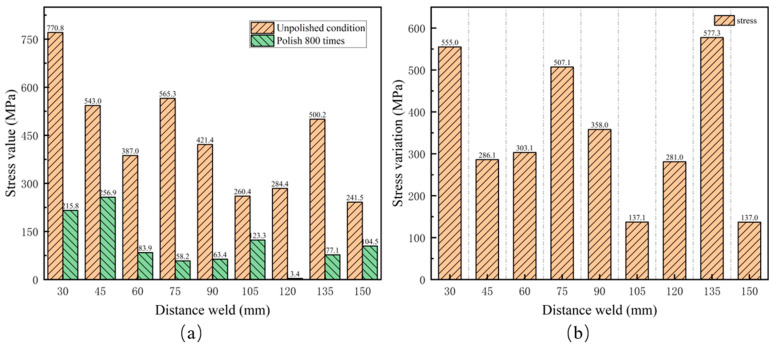
Residual stress test value and change of LCR before and after 800 times of grinding. (**a**) Residual stress measurements are unpolished and polished 800 times; (**b**) changes in residual stress after unpolished and polished 800 times.

Residual stress measurements were conducted on roughness samples in both unpolished state and after 800 polishing cycles using the LCR method, as shown in Figure 12a. The test results indicate that the residual stress values in the unpolished state are generally high. The minimum stress value is 241.5 MPa at a distance of 150 mm from the weld, while the maximum stress value reaches 770.8 MPa at 30 mm from the weld, significantly exceeding the yield strength (555 MPa) of X80 pipeline steel. This indicates that the stress measurement results under this surface condition are inaccurate. After 800 polishing cycles, the measured residual stress values decreased significantly. The maximum stress is 256.9 MPa, and the minimum stress is 3.4 MPa. The results also show that stress values are higher near the weld seam and lower further from the weld seam. Figure 12b shows the changes in stress measurement values after 800 polishing cycles compared to the unpolished state. At distances of 30 mm, 75 mm, and 135 mm from the weld, the changes in measurement values were 555.0 MPa, 507.1 MPa, and 577.3 MPa, respectively, all exceeding 500.0 MPa. Additionally, the changes at these positions were greater than the stress measurement values after 800 polishing cycles.

**Figure 13:**
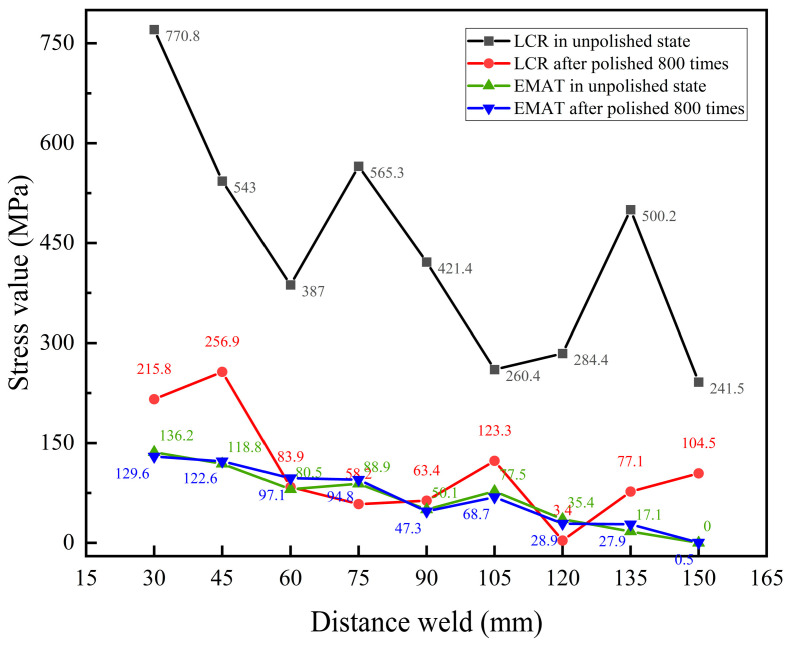
Comparison of EMAT and LCR stress measurements.

Figure 13 compares the stress measurement values between EMAT and LCR, both in the unpolished state and after 800 polishing cycles. The results indicate that the stress values measured by the EMAT in both states show minimal variation, with a maximum change of 25.5 MPa. This suggests that changes in surface roughness do not significantly affect the measurement accuracy. In contrast, the LCR wave method shows greater variations in stress values between the unpolished state and after 800 rounds of polishing. As shown in Figure 12b, at distances of 30 mm, 75 mm, and 135 mm from the weld seam, the variations are 555.0 MPa, 507.1 MPa, and 577.3 MPa, all exceeding 500.0 MPa. Especially in the unpolished state, the stress values at 30 mm, 45 mm, 75 mm, and 135 mm from the weld seam all exceed 500 MPa, approaching the yield strength of X80 pipeline steel. This suggests that surface roughness significantly impacts the stress measurement results of the critical refracted longitudinal wave method.

According to the stress detection theory of EMAT, EMAT waves primarily generate near the surface of the specimen and propagate along the thickness direction of the specimen. This process occurs internally within the sample, experiencing minimal influence from surface roughness. Additionally, as a non-contact measurement method, EMAT technology connects the transducer and specimen through electromagnetic coupling, eliminating the need for coupling agents to fill small gaps caused by surface roughness. Thus, it avoids reduced measurement accuracy due to poor acoustic coupling. Therefore, surface roughness may still affect the excitation energy of EMAT and the signal-to-noise ratio of the echo signal, which can influence the accuracy of stress measurement to some extent.

In contrast, the measurement principle of the LCR method indicates that LCR waves propagate along the near surface of the sample, and surface roughness significantly affects the propagation of LCR waves. The smaller the surface roughness, the greater the energy and amplitude of the LCR wave entering the specimen. When surface roughness is high, the effectiveness of acoustic coupling decreases, causing significant lateral scattering that reduces the directional focus of the LCR wave beam and lowers measurement sensitivity. Furthermore, a rough surface increases the propagation time of ultrasonic waves in the coupling agent, further impacting the accuracy of stress measurements.

The coating and surface roughness of pipelines significantly affect the accuracy of traditional ultrasonic testing methods. This study analyzed the coupling conditions of EMAT technology and experimentally investigated the effects of varying coating thicknesses and surface roughness on residual stress measurement. The results demonstrated that EMAT technology is less sensitive to surface conditions. This research validates the feasibility of the non-contact EMAT method for residual stress measurement without surface preparation and highlights its potential to significantly enhance pipeline inspection efficiency. These findings are of considerable importance for practical engineering applications.

## 5. Conclusions

This study investigated the effect of coating thickness and surface roughness on the accuracy of residual stress measurements using EMAT by making specimens with varying coating thicknesses and surface roughness. Based on the result, we can conclude the following:

(1) For specimens with coating thicknesses of 0.58 mm, 1 mm, 1.58 mm, and 1.9 mm, the stress measurement error compared to applied stress values remains in the range of 50 MPa. These indicate that the influence of the coating thickness on the measurement accuracy is negligible.
(2) The comparison of the stress measurements of roughness samples to unpolished samples revealed that the variation is less than 30 MPa, indicating that EMAT maintains good measurement stability across different surface roughness levels.
(3) Stress measurements of roughness specimens using LCR waves revealed that as surface roughness increased, the variation in stress measurement values significantly increased, highlighting the significant impact of surface roughness on the measurement accuracy.

EMAT can measure residual stress in pipelines without the need for surface treatment, thereby improving inspection efficiency. Thus, EMAT demonstrates significant potential for application in pipeline stress inspection and can effectively complement existing NDT techniques for pipelines.

In future research, the integration of electromagnetic acoustic transducer (EMAT) technology with the Finite Element Method (FEM) offers a promising approach for pipeline-integrity assessment. EMAT technology provides accurate and non-contact residual stress measurements, while FEM simulations can effectively predict future stress distributions and evaluate the performance of repair or reinforcement measures. Using EMAT-measured residual stress data as inputs or validation benchmarks for FEM models can significantly enhance the accuracy of stress analysis, enabling a more precise evaluation of the long-term reliability of pipelines. This combined approach is crucial for developing pipeline maintenance strategies and preventing crack propagation caused by residual stresses.

## Figures and Tables

**Table 1:** Surface roughness measurement results.

Coating thickness/times	0	40	80	120	160	200	400	800
$R_{a}$/μm	180.3	165.7	125.6	114.6	81.8	69.2	46.7	18.2

**Table 2:** Feedback amplitude of different coating thicknesses.

Coating thickness/mm	0	0.58	1	1.58	1.9
Echo amplitude/mV	481.1	320.2	215.1	109.4	98.7

**Table 3:** Stress test values for different coating thicknesses.

Coating thickness/mm	0	0.58	1	1.58	1.9
Load stress/MPa	Test stresses/MPa				
50	77.5	90.6	87.9	74. 6	89.3
100	91.5	140.1	138.1	120.1	82.6
150	132.0	190.5	179.9	173.7	147.3
200	184.7	216.1	206.8	223.1	171.8
250	230.1	251.7	225.6	267.8	275.3
300	304.7	346.6	260.1	270.4	307.2
350	343.7	366.8	346.2	309.3	363.1
400	420.0	398.9	376.6	388.3	413.6

## Data Availability

The original contributions presented in this study are included in the article. Further inquiries can be directed to the corresponding author.
